# Editorial: Molecular mechanisms underlying obesity and their links with other comorbidities

**DOI:** 10.3389/fmolb.2023.1334024

**Published:** 2024-01-25

**Authors:** Julio Plaza-Diaz, Ana I. Álvarez-Mercado, Bilian Yu, Rungroch Sungthong

**Affiliations:** ^1^ Department of Biochemistry and Molecular Biology II, School of Pharmacy, University of Granada, Granada, Spain; ^2^ Instituto de Investigación Biosanitaria ibs.GRANADA, Complejo Hospitalario Universitario de Granada, Granada, Spain; ^3^ Children’s Hospital of Eastern Ontario Research Institute, Ottawa, ON, Canada; ^4^ Institute of Nutrition and Food Technology, Biomedical Research Center, University of Granada, Armilla, Spain; ^5^ Department of Pharmacology, School of Pharmacy, University of Granada, Granada, Spain; ^6^ Department of Cardiovascular Medicine, The Second Xiangya Hospital, Central South University, Changsha, China; ^7^ FuRong Laboratory, Changsha, China; ^8^ Department of Biochemistry and Microbiology, Faculty of Pharmaceutical Sciences, Chulalongkorn University, Bangkok, Thailand

**Keywords:** obesity, diabetes, dysbiosis, cardiovascular disease, fatty liver disease, biomarker

Worldwide, obesity is becoming more prevalent and represents a major health concern for everyone (Khine et al., 2022). Obesity etiology involves many factors, including dietary, behavior, education, and genetic factors (Lin and Li, 2021). Some molecular mechanisms during adipocyte differentiation, such as the expression of transcription factors and the crosstalk between the protein kinase B (AKT)-glycogen synthase kinase-3β (GSK-3β) signaling pathway and the 5′-adenosine monophosphate-activated protein kinase (AMPK)-acetyl-CoA carboxylase (ACC) pathway, have been unveiled for their roles in governing obesity (Khine et al., 2022). Obesity is a well-recognized complex multifactorial disease that can pose several illness consequences, e.g., hyperglycemia, hyperlipidemia, hypertension, cardiovascular disease, and other less common comorbidities (e.g., infertility, aging, infectious diseases, and cancers) (Gutierrez-Cuevas et al., 2021; Khine et al., 2022). However, insights into the molecular mechanisms that link obesity and other comorbidities are still limited and need additional updates.

Adipose tissue is essential for metabolism. During development and weight gain, the ability of adipose tissue to grow is a prerequisite for successful adipogenesis (hyperplasia) or hypertrophy (expansion of pre-existing adipocytes). The extracellular matrix must be reorganized to provide adequate space for newly formed or enlarged adipocytes. In addition, it must ensure adequate vascularization and innervation. Consequently, impaired adipose tissue expandability leads to local hypoxia, tissue inflammation, insulin resistance, and adipocytic cell death. These pathological characteristics, particularly local insulin resistance, result in lipid spillover to organs, including the liver and skeletal muscles, eventually leading to systemic insulin resistance and metabolic syndrome (Ruiz-Ojeda et al., 2021). Several articles in this Research Topic provide a comprehensive overview of the molecular mechanisms underlying obesity. They also provide an overview of their involvement in other comorbid conditions.

As a dietary supplement in its disodium crystal form, pyrroloquinoline quinone (PQQ) is marketed as a natural ingredient in some foods. This compound has antioxidant and anti-inflammatory properties. As a result, PQQ significantly influences mitochondria function. Mitochondrial dysfunction has been associated with various health conditions, including obesity and obesity-related comorbidities. PQQ reduces visceral and hepatic fat accumulation in animal and cell culture studies. It may also enhance mitochondrial function and increase the number of mitochondria, improving lipid metabolism and inhibiting lipogenesis. In addition to reducing diet-induced obesity, PQQ alters the gut microbiota, which mitigates obesity risk, and improves obesity development in offspring through maternal supplementation. Among obesity comorbidities, PQQ ameliorates mitochondrial dysfunction and obesity-associated inflammation, thereby mitigating non-alcoholic fatty liver disease, chronic kidney disease, and type 2 diabetes (Mohamad Ishak and Ikemoto).

Non-alcoholic fatty liver and non-alcoholic steatohepatitis are types of non-alcoholic fatty liver diseases (NAFLD) that can progress to more severe stages (e.g., cirrhosis or liver cancer). NAFLD heterogeneity is based on cellular plasticity. This refers to a cell’s ability to acquire distinct identities or change its phenotype in response to environmental factors. Liver cells are classified as parenchymal or non-parenchymal, depending on their function. Hepatocytes and cholangiocytes are parenchymal cells, while Kupffer cells, hepatic stellate cells, and endothelial cells are non-parenchymal cells. Cells within this heterogeneous population adapt to environmental changes due to cellular plasticity. Following exposure to a variety of stimuli, such as fatty acids, inflammation, and oxidative stress, all such mentioned cells can exert diverse and complex responses at multiple levels during NAFLD progression (Park et al.). Exploring the molecular mechanisms underlying cellular lipid metabolism may further explain the bridge between obesity and NAFLD.

Dysbiosis, an imbalance in microbial homeostasis, is strongly associated with obesity-induced metabolic disorders such as type 2 diabetes. Intestinal leakage or the release of a diverse range of metabolites of microbes can disrupt metabolic homeostasis and potentiate chronic inflammation when gut microbial diversity is altered. Changing gut microbiota in response to obesity can worsen triacylglycerol and cholesterol levels. These substances regulate adipogenesis, lipolysis, and fatty acid oxidation. In addition to altering the microbiome and its metabolites, an intricate interaction between the gut-brain axis contributes to insulin resistance development via a disruption of bidirectional communication. Furthermore, obesity and type 2 diabetes are associated with a distinct microbial community within visceral adipose tissue. Mesenteric adipose tissue from patients with type 2 diabetes exhibited a specific bacterial signature. Recently, it has been demonstrated that gut-derived *Clostridium innocuum* migrates to the mesenteric adipose tissue and modulates its function by promoting M2 macrophage polarization, increasing adipogenesis and promoting microbial surveillance during Crohn’s disease. Taking these facts into account, prebiotics, probiotics, or fecal microbial transplantation may serve as effective means of managing type 2 diabetes in the gut and adipose tissue (Patra et al.).

An anti-diabetic compound derived from fungal metabolites and semi-synthetic derivatives, pyridylnidulin (PN), was found to induce glucose uptake. PN was tested in diet-induced obesity mice to determine its effects on liver lipid metabolism and anti-diabetic properties. Through dietary intervention with a high-fat diet for 6 weeks, male C57BL/6 mice were exposed to obesity as well as prediabetic conditions. In these obese mice, PN (40 or 120 mg/kg), metformin (150 mg/kg), or a vehicle were administered orally for 4 weeks. PN- and metformin-treated mice had improved glucose tolerance and lower fasting blood glucose levels. The PN and metformin groups had similar hepatic triglyceride levels regarding hepatocellular hypertrophy based on histopathological steatosis scores. PN (120 mg/kg)- and metformin-treated mice had decreased levels of plasma adipocytokines, such as tumor necrosis factor-alpha and monocyte chemoattractant protein-1. Furthermore, PN (120 mg/kg)- and metformin-treated mice showed significantly reduced expression of genes involved in lipid metabolism, including lipogenic enzymes. Additionally, phosphorylated AMP-activated protein kinase protein expression levels were increased in PN- and metformin-treated mice (Likitnukul et al.).

Miklosz et al. have evaluated the effects of three main factors, i.e., AS160 silencing, obesity, and metabolic syndrome, on lipid uptake and profile in fully differentiated adipocytes derived from mesenchymal stem cells (ADMSCs) of lean and obese postmenopausal women (with and without metabolic syndrome). Silencing AS160 did not significantly affect the cellular lipid profile of adipocytes derived from obese individuals. Comparing cells with the reference protein level, the authors observed no significant differences in triacylglycerols, diacylglycerols, and free fatty acids. As a result of AS160 knockdown, fatty acid oxidation was stimulated, which might indicate that adaptive mechanisms prevent excessive lipid accumulation. However, visceral adipocytes were insensitive to the intervention.

To this end, all contributions to this Research Topic offer impactful insights into the molecular mechanisms that mediate obesity and its comorbidities. However, additional Frontier research is needed to fill the knowledge gaps regarding the link between obesity and its associated diseases.
